# Le traitement anti-VEGF des néovaisseaux choroïdiens juxta-fovéolaires du fort myope: à propos d’une observation

**DOI:** 10.11604/pamj.2017.26.98.11049

**Published:** 2017-02-24

**Authors:** Moulay Omar Moustaine, Foued Dellali, Abbas El Husseini, Anne-Lise Hirsch

**Affiliations:** 1 Service d’Ophtalmologie, Hôpital 20 Août, CHU Ibn Rochd, Casablanca, Maroc; 2 Service d’Ophtalmologie, CH de Gonesse, Ile de France, France

**Keywords:** Forte myopie, Néovaisseau choroïdien, Anti-VEGF, High myopia, choroidal neovessel, anti-VEGF

## Abstract

Les néovaisseaux choroïdiens constituent une complication redoutable de la forte myopie rapportée dans 5 à 10% des cas. Ils doivent être pris en charge rapidement vu leur pronostic sombre. Les IVT des anti-VEGF constituent actuellement la nouvelle alternative thérapeutique dépassant de loin la thérapie photo-dynamique (PDT). Néanmoins l'algorithme thérapeutique anti-VEGF devant ce type de néovaisseaux reste un sujet de discussion entre les auteurs. A travers cette observation on essaie d'illustrer la difficulté de prise en charge de ces néovaisseaux et de discuter le schéma thérapeutique Anti-VEGF à suivre.

## Abstract (en)

Choroidal neovessels are a threatening complication of high myopia, accounting for 5 to 10% of cases. They require immediate treatment because of their poor prognosis. Anti-VEGF intravitreal injections are currently a new therapeutic alternative far exceeding photodynamic therapy (PDT). Nevertheless, anti-VEGF treatment algorithm for this type of neovessels remains a matter of discussion among the authors. The purpose of this study was to highlight the difficulties in managing these neovessels and to discuss the Anti-VEGF therapeutic regimen to follow.

## Introduction

Les néovaisseaux choroïdiens (NVC) constituent une complication redoutable de la forte myopie, ils surviennent chez 5 à 10% des forts myopes et doivent être pris en charge rapidement [1, 2]. Les injections intravitréennes (IVT) des anti-VEGF constituent actuellement une nouvelle alternative thérapeutique dépassant de loin la photothérapie dynamique (PDT) [3], avec une efficacité confirmée par plusieurs études qui montrent des résultats anatomiques et fonctionnels prometteurs [4, 5]. L'algorithme thérapeutique des IVT d'anti-VEGF devant un NVC du fort myope reste actuellement un sujet de discussion entre les auteurs surtout en absence de bénéfice de la phase d'induction initiale comme dans la DMLA [6–8]. A travers une observation d'un NVC de fort myope ayant bien évolué sous traitement anti-VEGF on essaie d'illustrer la difficulté de prise de ce type de NVC et discuter les modalités thérapeutiques.

## Patient et observation

**Figure 1:**
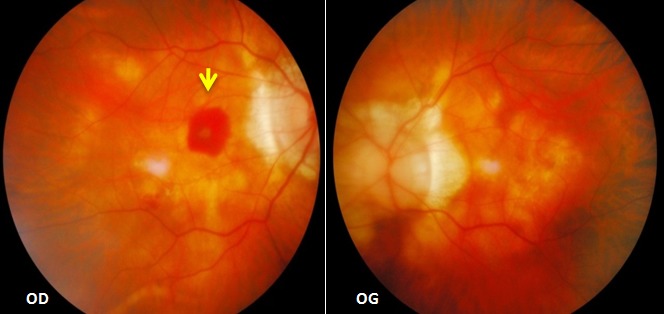
Rétinophotographie couleur de l’OD et de l’OG à l’admission du patient: (présence au niveau de l’OD d’une hémorragie intrarétinienne profonde localisée en périfoviolaire; choroïdose myopique manifeste ODG)

**Figure 2:**
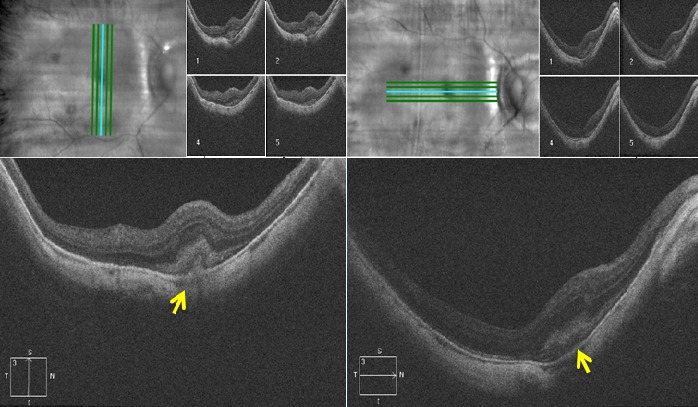
OCT de l’OD à l’admission du patient (SD-OCT CIRRUS 4000 HD) : (hyper réflectivité fusiforme en avant du plan membrane du Bruch-EP en rapport avec un néovaisseau choroïdien pré-épithélial visible (type II) ; absence de phénomène exsudative, de DSR ou de drusen)

**Figure 3:**
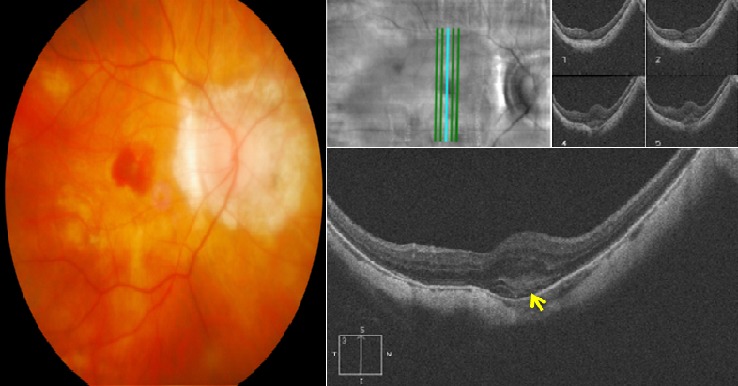
Rétinophoto et OCT de l'OD un mois après la première IVT d’anti-VEGF (SD-OCT CIRRUS 4000 HD): (rétinophoto: régression de la taille de l'hémorragie rétinienne; OCT: régression de l'hyper-réflectivité fusiforme en rapport avec le néovaisseau avec toujours absence de phénomènes exsudatives)

**Figure 4:**
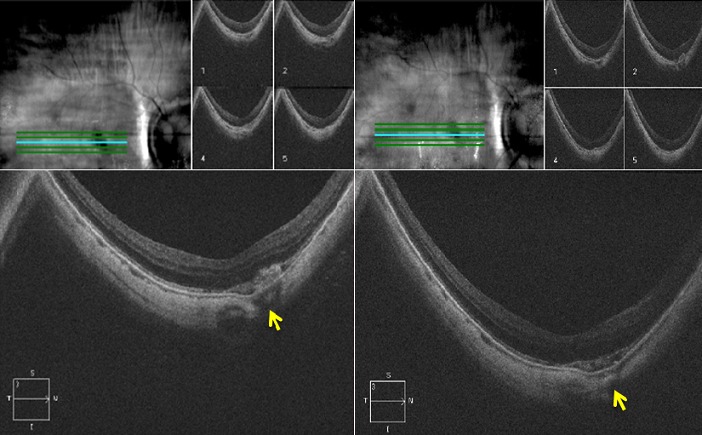
OCT de l'OD un mois après l'IVT de renforcement (SD-OCT CIRRUS 4000 HD): (noter la nette régression de la taille de l'hyper- réflectivité fusiforme en rapport avec le néovaisseau)

**Figure 5:**
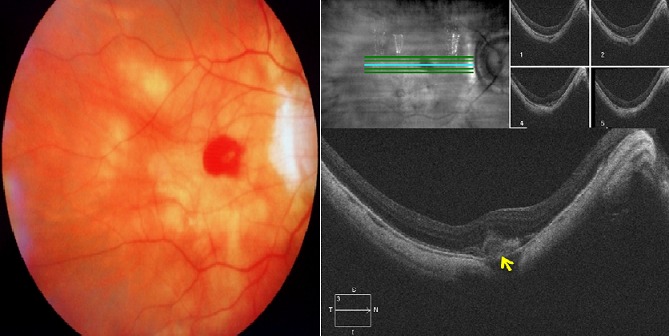
Rétinophoto et OCT de l'œil droit 3 mois après l'IVT de renforcement (SD-OCT CIRRUS 4000 HD): (rétinophoto: réactivation des néovaisseaux avec resaignement au même endroit; OCT: augmentation de nouveau de l'hype-réflectivité fusiforme, avec apparition d'une petite lame de DSR (flèche))

**Figure 6:**
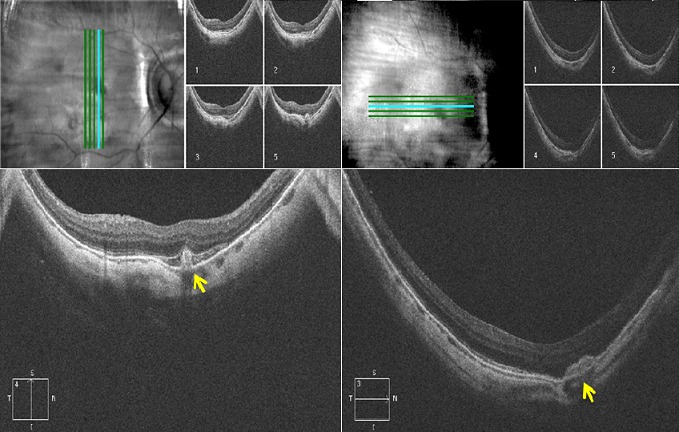
OCT de l'OD un mois après la deuxième IVT de la série de trois (SD-OCT CIRRUS 4000 HD): (nette régression de la taille de l'hyper- réflectivité fusiforme en rapport avec le néovaisseau visible)

**Figure 7:**
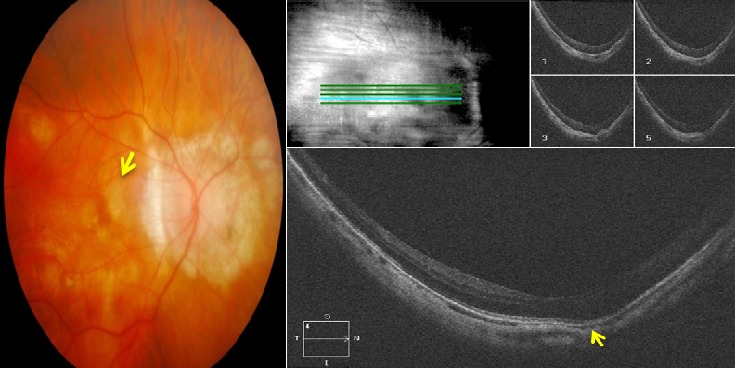
Rétinophoto et OCT de l'OD, 6 mois après la dernière IVT de la série de trois (SD-OCT CIRRUS 4000 HD): (rétinophoto : résorption totale de l'hémorragie rétinienne périfoviolaire; OCT : normalisation quasi-complète du profil maculaire avec disparition de l'hyper réflectivité fusiforme et du lame de DSR)

Il s'agit de monsieur T.M, âgé de 78 ans, fort myope et pseudophaque ODG, sans autres antécédents pathologiques notables. Le patient consulte aux urgences d'ophtalmologie du centre hospitalier de Gonesse pour un scotome central unilatéral de l'OD d'apparition brutale avec BAV d'aggravation progressive depuis une semaine. À L'examen ophtalmologique, les annexes, l'oculomotricité ainsi que les réflexes photo-moteurs direct et consensuel sont normaux. Au niveau de l'OD, l'AV corrigée de loin et de prés est très basse : 1/10 P16, le segment antérieur est normal avec un implant cristallinien clair et en place, le tonus oculaire est à 14 mmHg. Au fond d'œil (FO), on note la présence d'une hémorragie intrarétinienne périfoviolaire profonde et une choroïdose myopique diffuse (Figure 1). Au niveau de l'œil gauche, l'AV corrigée est conservée : 6/10 P2.5, le segment antérieur est normal, l'implant cristallinien est clair en place, le tonus oculaire est à 13 mmHg. Le FO est normal en dehors d'une choroïdose myopique diffuse (Figure 1). L'OCT de l'OD réalisée en urgence montre la présence d'une hyper-réflectivité fusiforme périfoviolaire en avant du plan de la membrane du Bruch-épithélium pigmentaire (EP) en rapport avec un néovaisseau choroïdien pré-épithélial visible (type II) sans phénomènes exsudatives ni drusen associés (Figure 2). Notre conduite thérapeutique était de suivre une stratégie « 1+PRN » en réalisant une IVT d'Anti-VEGF (ranibizumab) avec un suivie rigoureux (AV, FO, rétinophotos et l'OCT), tout en informant le patient sur le pronostic de l'affection et les signes qui doivent l'amener à consulter sans délais. Le control un mois après montre une amélioration de l'AV de l'OD : 2/10 P9, avec au FO une régression de la taille de l'hémorragie périfoviolaire. A l'OCT on note également une régression de l'hyper-réflectivité fusiforme périfoviolaire sans phénomènes exsudatifs (Figure 3). Devant cette amélioration partielle, le patient a bénéficié d'une deuxième IVT d'Anti-VEGF (ranibizumab) de renforcement en assurant toujours une surveillance mensuelle. Un mois plus tard l'AV continue à s'améliorer : 3/10 P5, avec au FO et en OCT une nette régression de la taille de l'hémorragie et du néovaisseau (Figure 4), ce résultat nous a amené à continu la surveillance sans réalisation d'autres IVT. Deux mois plus tard, le patient consulte en urgence pour rebaisse de l'AV. L'examen ophtalmologique retrouve une rechute de l'AV de l'OD à 1/10P16, avec au FO un resaignement pérfoviolaire et à l'OCT une augmentation de nouveau de l'hyper-réflectivité fusiforme pérfoviolaire évoquant une réactivation du même NVC (Figure 5). Devant ce tableau on a optée d'emblé à la réalisation d'une série de trois IVT d'anti-VEGF, d'un mois d'intervalle en assurant une surveillance mensuelle stricte. L'évolution au cours puis après cette série d'IVT est favorable, marquée sur le plan fonctionnel par une régression du scotome central et amélioration progressive de l'AV (4/10) P9 et sur le plan clinique une résorption totale de l'hémorragie pérfoviolaire sur une période de quatre mois. A l'OCT la résorption de l'hyper-réflectivité fusiforme périfoviolaire était très progressive jusqu'a disparition quasi-totale six mois après la dernière IVT (Figure 6, Figure 7).

## Discussion

La myopie est la première étiologie des néovaisseaux survenant chez les sujets de moins de 50 ans [3], ces néovaisseaux sont rapporter chez 5 à 10% des forts myopes [1, 2], leur évolution spontanée est défavorable et marquée par la constitution d'une cicatrice fibrovasculaire pigmenté (tache de Fuchs), remplaçant la choriocapillaire et les couches externes de la rétine [4]. Les traitements antérieurs proposés sont la photo coagulation au laser Argon supplantée dans les années 2000 par la photothérapie dynamique (PDT) [5, 6]. Les anti-VEGF constituent actuellement le traitement de première intention, leur efficacité est bien établie ainsi que leur nette. L'algorithme thérapeutique et le nombre des IVT nécessaires restent un sujet de discussion entre les auteurs. Certes plus faible que la DMLA avec absence de bénéfice de la phase d'induction (série de trois IVT d'emblé au début), car le NVC du fort myope constitue un phénomène aigu, focal et souvent associé à une rupture de la membrane de Brüch alors que dans la DMLA reste une maladie chronique, diffuse et nécessite plus de retraitements [6, 10]. Certains auteurs recommandent une stratégie thérapeutique type « 1+PRN » avec la réalisation d'une seule IVT à la découverte du NVC suivie des retraitements à la demande en fonction de l'activité néovasculaire. Devant un NVC de grande taille ou très exsudatif ils recommandent de commencer d'emblée par une phase d'induction suivie d'une stratégie PRN [4, 10]. Au total devant un NVC du fort myope le schéma thérapeutique Anti-VEGF à suivre doit être bien guidé, avec un suivie clinique (AV et FO) et para clinque (Rétinophotos, OCT) rigoureux. Le patient doit être averti de la gravité de son affection et doit être revu de façon régulière afin de dépister tôt toute réactivation néovasculaire et décider un éventuel retraitement au bon moment. Ces retraitements sont d'autant plus efficaces qu'ils sont réalisés précocement avant que la rétine soit définitivement endommagée [4, 6, 10].

## Conclusion

Le schéma thérapeutique anti-VEGF devant un NVC du fort myope reste difficile à standardiser. Les algorithmes thérapeutiques proposés à ce jour sont basés essentiellement sur les connaissances théoriques et l'expérience pratique acquise. Les auteurs recommandent la réalisation d'une seul IVT d'anti-VEGF au début suivie d'une stratégie «PRN» dont les retraitements simples ou renforcés reposent sur l'importance de l'activité néovasculaire [6, 9, 10]. Des études de grande échelle sont actuellement nécessaires pour établir un schéma thérapeutique validé devant cette affection grave et cécitante.
